# Patterns of change in obesity indices and other cardiometabolic risk factors before the diagnosis of type 2 diabetes: two decades follow-up of the Tehran lipid and glucose study

**DOI:** 10.1186/s12967-022-03718-8

**Published:** 2022-11-08

**Authors:** Fatemeh Koohi, Nooshin Ahmadi, Fereidoun Azizi, Davood Khalili, Majid Valizadeh

**Affiliations:** 1 Obesity Research Center, Research Institute for Endocrine Sciences, Shahid Beheshti University of Medical Sciences, Tehran, Iran; 2 Prevention of Metabolic Disorders Research Center, Research Institute for Endocrine Sciences, Shahid Beheshti University of Medical Sciences, Tehran, Iran; 3 Endocrine Research Center, Research Institute for Endocrine Sciences, Shahid Beheshti University of Medical Sciences, Tehran, Iran

**Keywords:** Diabetes mellitus, Obesity indices, Cardiometabolic risk factors, Multivariate latent class growth, Mixed model, Linear mixed effect model

## Abstract

**Background:**

Identifying patterns of variation in obesity indices and other cardiometabolic risk factors before the diagnosis of type 2 diabetes could provide insight into the critical period when drastic changes occurred and facilitate targeted interventions for the prevention of diabetes. Therefore, this study sought to explore patterns of change in obesity indices and other cardiometabolic risk factors before diabetes diagnosis.

**Methods:**

We investigated 6305 participants (43.7% men) aged 20–65 from the Tehran Lipid and Glucose Study (TLGS) who were free of diabetes at baseline. First, we jointly estimated developmental multi-trajectories of obesity indices using multivariate latent class growth mixed model, and then patterns of cardiometabolic risk factors within the identified multi-trajectories were assessed using mixed-effects models.

**Results:**

Three patterns of change in obesity indices were identified. Most participants belonged to the “progressing” group (83.4%; n = 742), with a slight but steadily rising in obesity indices until diagnosis in both men and women. All multi-trajectory groups showed similar exponential increases in fasting and 2-h plasma glucose concentrations 6 years before diagnosis and linear increases in blood pressure and total and LDL cholesterol throughout follow-up. Patterns of triglyceride and HDL cholesterol accompanied each group’s patterns of change in obesity indices.

**Conclusion:**

Three patterns of the joint progression of obesity indices before diabetes diagnosis were accompanied by similar blood glucose patterns and other cardiometabolic risk factors. These findings suggest the impact of the increasing trend of obesity indices and other metabolic factors on the incidence of diabetes and emphasize the importance of assessing the metabolic risk factors at each visit.

**Supplementary Information:**

The online version contains supplementary material available at 10.1186/s12967-022-03718-8.

## Introduction

Diabetes remains a growing health challenge worldwide [1]. People with diabetes are at risk for many life-threatening health problems that increase the cost of medical care, reduce the quality of life, and increase mortality [2]. The prevalence of diabetes has risen rapidly in many countries and regions, along with rapid urbanization and dramatic lifestyle changes [1, 3]. The International Diabetes Federation (IDF) estimated the global prevalence to be 451 million in 2017 and is expected to increase to 693 million by 2045 [4]. It was also projected that approximately half of all people living with diabetes are undiagnosed [4].

Obesity is the main modifiable risk factor for type 2 diabetes. Some evidence suggests that the degree of obesity and where fat accumulates determine the influence of obesity on the risk of developing type 2 diabetes; however, it is unclear how different obesity indices vary during the natural history of progression to diabetes. Such evidence could help further to facilitate targeted prevention programs for diabetes and its complications [5]. Few studies have investigated longitudinal changes (referred to as trajectories) of various indices of obesity [6–8]. However, these studies considered the trajectory of each obesity indices separately, ignoring the correlation between different indexes that do not account for the fact that these indices may measure the same underlying quantity.

In addition, other cardiometabolic risk factors, from plasma glucose levels to elevated blood lipids, and hypertension, also predispose individuals to cardiovascular disease (CVD) and type 2 diabetes [9–11]. A few studies have reported trajectories of hypertension, blood glucose, and lipid profile in adults with diabetes [5, 8, 12]; however, little is known about the dynamic trends in cardiometabolic risk factors accompanying different patterns of obesity indices before the onset of diabetes.

Identifying patterns of variation in obesity indices and other cardiometabolic risk factors before the diagnosis of type 2 diabetes could provide insight into the critical period when drastic changes occurred and facilitate targeted interventions for the prevention of diabetes. One way of better understanding intra- and inter-individual variability in health outcome patterns over time and identifying subgroups (distinct trajectories) representing similarities in given outcomes is to use trajectory modeling approaches [13, 14]. To the best of our knowledge, no previous study has been reported to jointly investigate the patterns of obesity indices and trajectories of other cardiometabolic risk factors accompanied with them in a population initially free of diabetes.

Using repeated measurements of cardiometabolic risk factors in a longitudinal cohort from an Iranian population, we sought to jointly explore common latent classes and patterns of obesity indices in a population initially free of diabetes based on multivariate trajectory analysis. Then, we examined trajectories of other metabolic risk factors accompanying each distinct multi-trajectory of obesity development before diagnosing diabetes.

## Methods

### Study design and population

We used data from the Tehran Lipid and Glucose Study (TLGS), an ongoing prospective population-based cohort study among a representative sample of an urban Iranian population. Details regarding the methods and design of TLGS have been reported previously [15]. Briefly, 15,005 men and women aged 3 years and more living in Tehran, district No. 13, were recruited in the first examination of the study in 1999–2002. The participants of TLGS have been followed up on a triennial basis, and to date, the data have been collected across six consecutive examinations. The study protocol was approved by the Ethics Committee of the Research Institute for Endocrine Sciences, Shahid Beheshti University of Medical Sciences.

Since the trajectory approach requires at least three unique time points to identify nonlinear patterns, the current study was restricted to participants attending at least three examinations. Of 11,611 participants aged 20–65 years who attended the first (n = 9356) or second (n = 2255) examinations as the baseline assessment, we excluded those with type 2 diabetes (n = 1092), and missing information on diabetes status (n = 967) at baseline. In addition, we excluded women who were pregnant in each examination (198). Besides, we excluded participants who did not receive any glucose measurement after recruitment until the end of the study (n = 1729) as we could not ascertain their diabetes status and those who had less than three nonmissing obesity measurements available (n = 1320). Finally, 6305 participants followed up until April 2018 and remained in the trajectory analysis.

### Assessment of cardiometabolic indices and covariates

During all study examinations, anthropometric measurements, including weight, height, and waist circumference (WC), were measured according to standard protocols in standing with light clothing and no shoes. Body mass index (BMI) was calculated as weight in kilograms divided by height in square meters. Systolic and diastolic blood pressure were measured as the average of two measurements in the sitting position at 5-min resting intervals. All baseline and follow-up biochemical measurements, including fasting plasma glucose (FPG), 2-h plasma glucose (2-hPG), and all blood lipid analyses, including total cholesterol (TC), low-density lipoprotein cholesterol (LDL-C), high-density lipoprotein cholesterol (HDL-C), and triglycerides (TG) were collected according to standardized procedures using peripheral blood samples collected after a 12-h overnight fast and 2-h after glucose ingestion. Data on demographic, smoking status, physical activity, past medical history, and family history of diabetes were collected via standard questionnaires at each examination. Smoking status was categorized as current smokers who smoked cigarettes or tobacco products daily or occasionally versus nonsmokers who never smoked or smoked in the past. Physical activity was classified as low and high, defined as a score of less than 600 and equal to or more than 600 MET-minutes per week. Family history of diabetes was described as having diabetes in first-degree relatives, including parents or children. Based on the American Diabetes Association (ADA) criteria [16], diabetes was defined as either FPG ≥ 7 mmol/L, a 2-hPG ≥ 11.1 mmol/L, or receiving antidiabetic medication, and prediabetes was defined as FPG of 5.6–6.9 mmol/L or 2-hPG of 7.8–11.0 mmol/L.

### Statistical analysis

Characteristics between subgroups were compared using the chi-square test for categorical variables, t-tests for normal continuous data, and Mann–Whitney U test for non-normal continuous data.

Retrospective multi-trajectories of obesity indices were modeled using a backward timescale. The observation period started (year 0) at the examination when diabetes was first identified for participants who developed diabetes and at the last examination or lost to follow-up for those who did not develop diabetes. Participants were then followed back to the baseline examination.

We used a multivariate latent class growth mixed model with BMI, waist, and waist-to-hip ratio as dependent variables to explore the joint heterogeneity of obesity indices in the population developing diabetes. A series of polynomial specifications of obesity indices as a function of time before diabetes diagnosis with a class number ranging from 1 to 5 were assessed in the population developing diabetes using the multlcmm function of the “lcmm” (version 1.9.3) package in R Foundation for Statistical Computing, Vienna, Austria. version 4.0.3) [17]. An outcome-specific random intercept and the random slope were considered in the modeling process. A linear term for the time before diagnosis was used to specify the random effects of the model, i.e., the individual variation around the average change over time (fixed effect). When significant, quadratic and cubic terms for the time were included in the models for individuals who developed diabetes. The best fit model with the optimal number of latent classes was selected by the following criteria: [1] the least Bayesian information criterion (BIC); a reduction of BIC of at least ten points; [2] a posterior probability above 0.7 for all latent classes; and [3] no less than 5% of the diabetes population in any single trajectory class.

After identifying the latent classes of the obesity indices under study, we next assigned diabetes participants exclusively to the class for which the highest posterior probability of membership was obtained upon the model fit. To examine the trajectories of the accompanying cardiometabolic risk factors, including fasting and 2-h plasma glucose; systolic and diastolic blood pressure; total, LDL and HDL cholesterol, and triglycerides, linear mixed-effects models were implemented using the lme function of the “nlme” (version 3.1-153) package in R version 4.0.3 [17]. The linear mixed model can handle any unbalanced data, including unequal numbers and time of measurements per subject, correlation of repeated measures within subjects, and missing at-random data [18].

For individuals who developed diabetes, quadratic and cubic terms for the time were added to the model allowing for nonlinear developmental patterns of cardiometabolic risk factors when significant (P-value < 0.05). Still, for individuals who did not develop diabetes, we fitted the trajectories by linear terms for time, as year 0 was merely a time point in an ordinary life course. Still, for individuals who did not develop diabetes, we fitted a one-class model by linear terms for time, as year 0 was merely a time point in an ordinary life course.

Pairwise curve differences between the obesity subgroups were tested using the F test by comparing the curve of contrasts with a straight line with zero slopes. Therefore, provided p values are related to differences in slope, intercept, or both.

Differences in the latent classes and trajectories between men and women were tested using standard Wald tests. All analyses were adjusted for age, sex, and study phase; blood pressure multi-trajectories were further adjusted for antihypertensive treatment, and trajectories of lipids were further adjusted for the lipid-lowering treatment. A two-sided P < 0.05 was considered statistically significant.

## Results

### Patterns of obesity indices development

A total of 6305 participants (2755 men) aged 20–65 were included in the current study. Additional file 1: Fig. S1 presents the participants’ flowchart in the current study. On average, participants had 3.3 (range, 3–6) times obesity indices measurements. During a median follow-up of 15.6 years (range 4.6–19.1), 883 incident diabetes cases were identified. Of these, 374 (42.4%) were men, and the mean (SD) age at baseline was 43.4 (10.9) years.

According to the criteria mentioned above, a model of cubic terms for the time before diabetes diagnosis with three distinct patterns of BMI, WC, and WHR development was chosen from all investigated models. The latent class growth mixed model results of the fitting process and detailed parameter estimates of the best fitting 3-class cubic trajectory are shown in Additional file 2: Table S1 and Additional file 3: Table S2. The average posterior probability of class membership for individuals was high for each class (80–91%) (Additional file 2: Table S1). Sex term was insignificant, indicating that men and women did not have different patterns of obesity indices. However, its interaction with time and contrast terms was significant, showing significantly differential effects of sex on obesity indices (p = 0.00001) with a systematically higher BMI for women and higher waist-to-hip ratios for men over time (Additional file 3: Table S2).

**Fig. 1:**
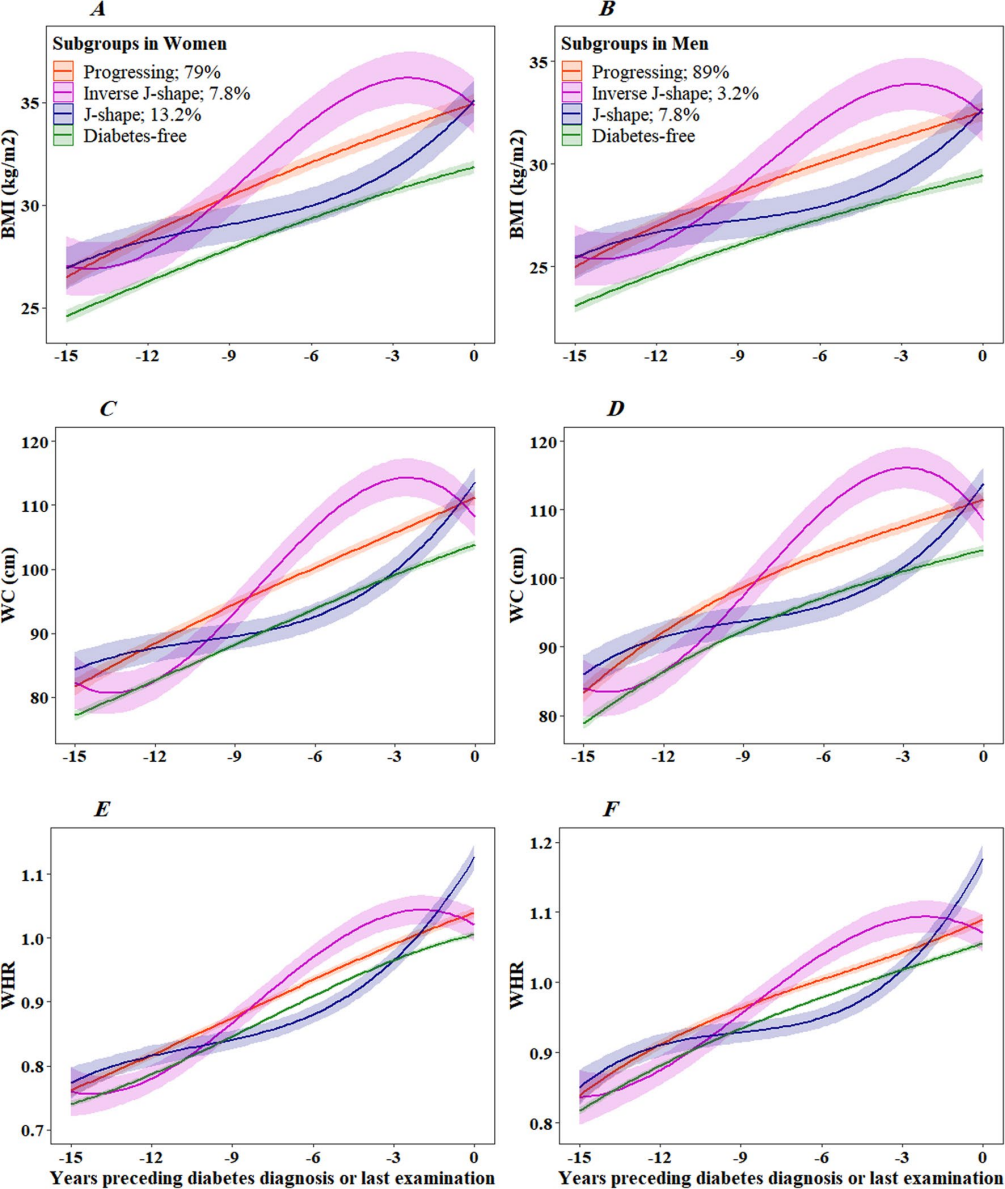
Multi-trajectories of body mass index (**A**, **B**), waist circumference (**C**, **D**), and waist-to-hip ratio (**E**, **F**) for women and men of 53 years of age at time 0 from 15 years before the diagnosis of type 2 diabetes or last examination. Lines are the estimated trajectories, and shadows are 95% CIs

Figure 1 shows the predicted mean trajectory of obesity indices in women and men. Three trajectories were labeled as progressing, representing the majority of individuals who developed diabetes (83.4%; n = 742), inverse J-shape (5.8%; n = 52), and J-shape (10.8%; n = 96).

For progressing class, the predicted trajectory of obesity indices rose slightly but steadily until diagnosis in both men and women. The mean BMI in women was in the obese range from 10 years before diagnosis, whereas in men, it was in the overweight range until 7–6 years before diagnosis. The average weight gain in women and men was 8 and 7.5 BMI units during 15 years of follow-up, respectively. Both WC and WHR were in the central obese range based on national cut-off points (WC ≥ 90 cm in both sexes, WHR ≥ 0.90 in men, and WHR ≥ 0.85 in women) from around 11 and 13 years before the diagnosis of diabetes in women and men, respectively. During 15 years of follow-up, an average increase of 29.6 and 28.2 BMI units, 0.28 and 0.25 WHR was observed in women and men, respectively.

In the inverse J-shape class, the predicted trajectory of obesity indices peaked around 3 years before diagnosis and then started to fall; all obesity indices were in the obese range around 10 years before diagnosis. This group had an average increase of 7.7 and 6.8 kg/m^2^ in BMI; 25.9 and 24.5 cm in WC units; 0.26 and 0.23 units in WHR during the 15 years of follow-up, respectively, in women and men.

In the J-shape class, the predicted trajectory of obesity indices was slightly increasing from 15 to around 6 years before diagnosis by an average increase of 2.7 and 2.2 BMI units; 6.8 and 8.9 WC units, 0.09 in WHR, respectively, in women and men. After that, obesity indices rose steeply until the time of diagnosis. This group had an average increase of 5.5 and 5.1 kg/m^2^ in BMI; 22.5 and 19.0 cm in WC units; 0.27 and 0.24 units in WHR 6 years before diagnosis, respectively, in women and men.

The average BMI, WC, and WHR development in the reference group not developing diabetes was 7.2 and 6.3 BMI units; 26.7 and 25.3 WC units; 0.26 and 0.23 WHR during the 15 years of follow-up in women and men, respectively.

Table 1 summarizes the baseline characteristics of the study population by obesity indices trajectory classes. Compared with the diabetes-free population, individuals who developed diabetes were more likely to have a family history of diabetes, and higher values of FPG, 2hPG, BMI, SBP, DBP, and TG. Besides, the progressing class had higher WC, TC, and LDL-C values and lower HDL-C than the diabetes-free group.

**Table 1:** Baseline characteristics of study participants who did and did not develop diabetes at follow-up

Characteristics	Individuals developing diabetes in the study stratified by latent classes of obesity indices			Individuals Free of diabetes (n = 5422)
	Progressing (n = 735 )	Inverse J-shape (n = 52 )	J-shape (n = 96)	
Time before diabetes diagnosis/last examination, years	15.9 (2.0)	16.1 (1.9)	15.8 (1.6)	15.6 (2.3)^a^
Men (%)	333 (45.3)	12 (23.1)	29 (30.2)	2381 (43.9)^d^
Age, years	43.8 (10.8)	41.3 (12.4)	41.4 (11.3)	38.2 (11.6)^a,c^
BMI (kg/m^2^)	29.0 (4.7)	28.9 (4.9)	28.7 (4.5)	26.2 (4.3)^a,b,c^
WC (cm)	94.2 (11.1)	89.7 (11.6)^a^	91.8 (11.0)	86.1 (11.4)^a,c^
Family history of diabetes	268 (37.1)	19 (36.5)	37 (40.2)	1309 (24.8)^d^
FPG (mmol/L)	5.3 (0.6)	5.2 (0.6)	5.2 (0.6)	4.9 (0.5)^a,b,c^
2-hPG (mmol/L)	6.8 (1.7)	6.6 (1.5)	6.7 (1.7)	5.6 (1.4)^a,b,c^
TC (mmol/L)	5.7 (1.2)	5.5 (1.1)	5.6 (1.2)	5.2 (1.1)^a,c^
LDL-C (mmol/L)	3.7 (0.9)	3.4 (0.9)	3.5 (0.9)	3.3 (0.9)^a^
HDL-C (mmol/L)	1.0 (0.2)	1.1 (0.3)	1.1 (0.2)	1.1 (0.3)^a^
TG (mmol/L)	2.3 (1.2)	2.2 (1.0)	2.4 (1.6)	1.7 (1.1)^a,b,c^
Lipid-lowering drug	26 (3.5)	2 (3.8)	3 (3.1)	93 (1.7)^d^
SBP (mmHg)	122.4 (17.3)	121.5 (17.8)	120.5 (16.4)	114.5 (15.3)^a,b,c^
DBP (mmHg)	80.9 (10.1)	80.7 (11.1)	80.2 (10.1)	75.8 (10.1)^a,b,c^
Antihypertensive drug	65 (8.8)	8 (15.4)	7 (7.3)	179 (4.1)^a,b,c^
Current smoking	94 (12.8)	4 (7.7)	11 (11.6)	727 (13.4)

Data are n (%), mean (SD), or median (IQR)

*FPG* fasting plasma glucose, *2-hPG* 2-hour plasma glucose, *BMI* body mass index, *WC* waist circumference, *TC* total cholesterol, *LDL-C* low-density lipoprotein cholesterol, *HDL-C* high-density lipoprotein cholesterol, *TG* triglycerides, *SBP* systolic blood pressure, *DBP* diastolic blood pressure

^a^Significantly different from progressing

^b^Significantly different from Inverse J-shape

^c^Significantly different from J-shape

^d^Significantly different from diabetes subgroups

### Trajectories of plasma glucose, blood pressure, and lipids

**Fig. 2:**
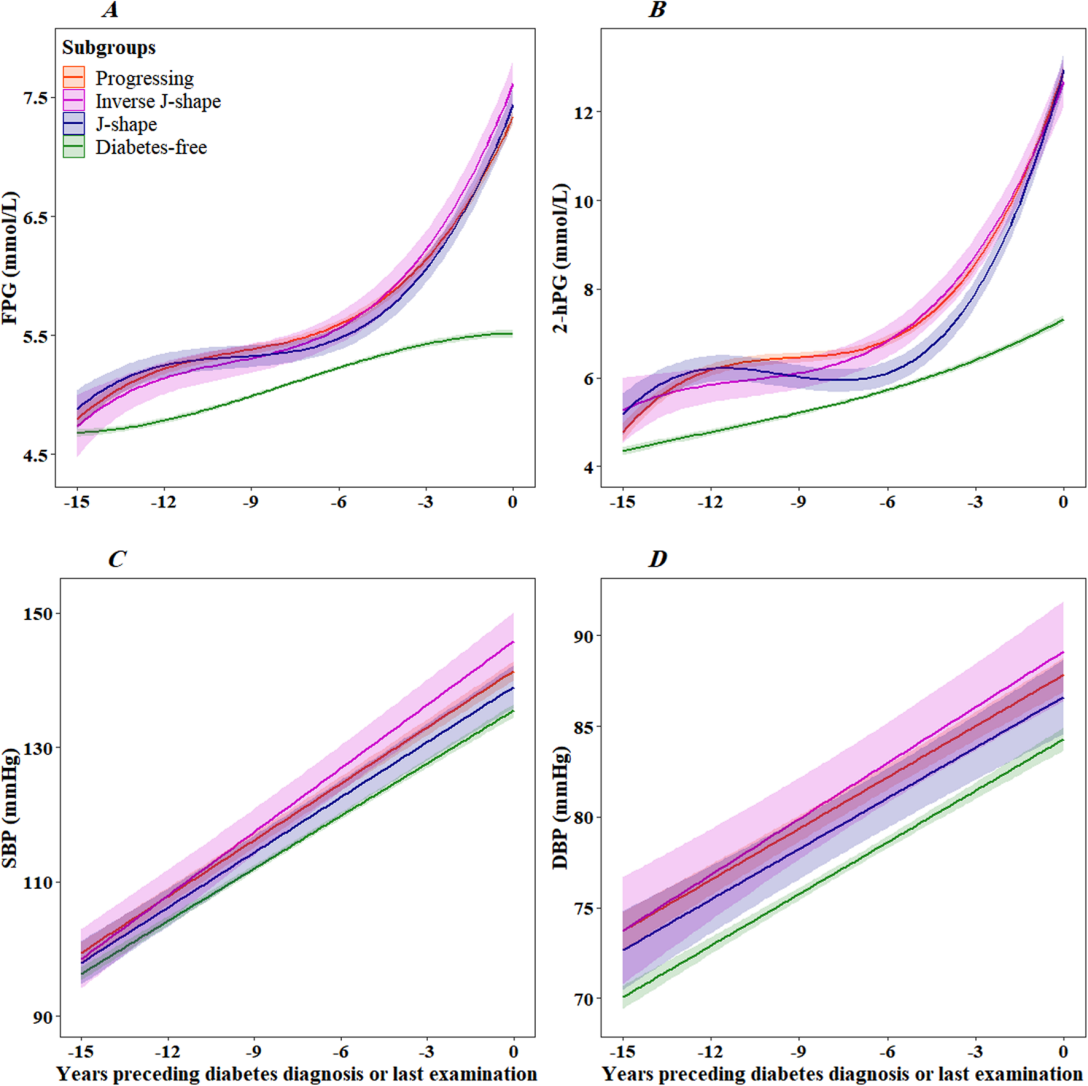
Trajectories of fasting plasma glucose (**A**), 2-h plasma glucose (**B**), systolic blood pressure (**C**), and diastolic blood pressure (**D**) for men of 53 years of age at time 0 from 15 years before the diagnosis of type 2 diabetes or last examination. Trajectories for blood pressure represent men, not antihypertensive treatment. Lines are the estimated trajectories, and shadows are 95% CIs

Trajectories of fasting and 2-h plasma glucose concentrations were similar in all three groups with a slightly but steadily rise from 15 years before diagnosis until around 5 to 6 years before diagnosis when both FPG and 2-hPG concentrations rose exponentially towards diabetes diagnosis (Fig. 2A, B, p > 0.05 for all). Individuals not developing diabetes exhibited normal FPG and 2-hPG concentrations during 15 years of follow-up.

Trajectories of systolic blood pressure showed a linearly rising pattern toward diabetes diagnosis in the three subgroups. We found differences in SBP trajectories in the inverse-J-shape group compared with the others (Fig. 2C, P < 0.05 for both). However, the SBP trajectory did not differ between the progress and J-shape groups (Fig. 2C, P = 0.118). In general, the trajectory of diastolic blood pressure followed those of SBP; however, the DBP trajectory was in the normal range during follow-up. It did not differ significantly (Fig. 2D, p > 0.05 for all).

**Fig. 3:**
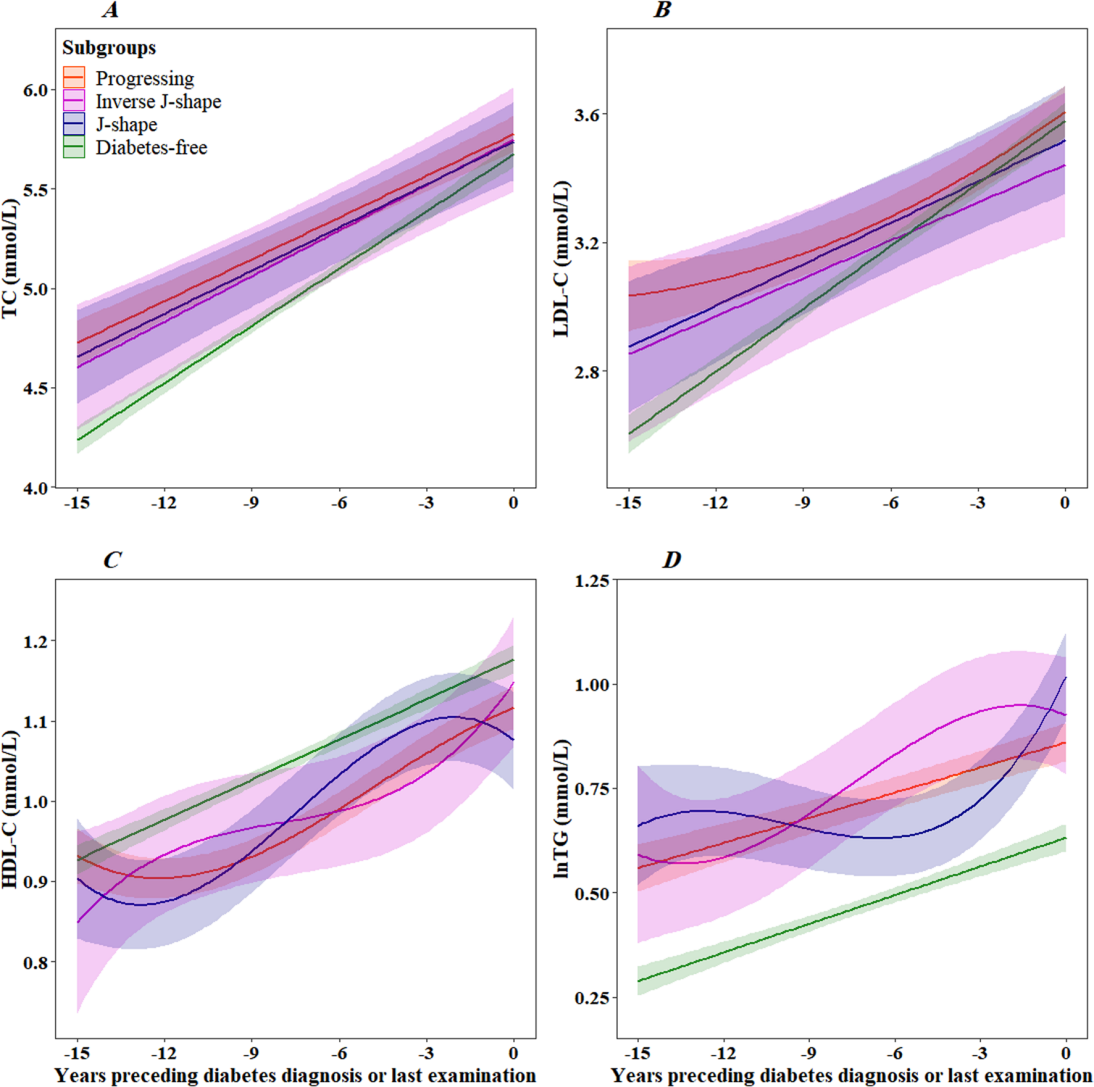
Trajectories of total cholesterol (**A**), low-density lipoprotein cholesterol (**B**), high-density lipoprotein cholesterol (**C**), and the logarithm of triglycerides (**D**) concentrations for men of 53 years of age at time 0 and not on lipid-lowering treatment from 15 years before the diagnosis of type 2 diabetes or last examination. Lines are the estimated trajectories, and shadows are 95% CIs

The total and LDL cholesterol concentrations showed a linearly increasing pattern toward diabetes diagnosis in all groups. The predicted TC and LDL-C levels trajectories were in borderline and high ranges during 15 years of follow-up. They did not differ significantly between subgroups (Fig. 3A, B, P > 0.05 for all pairwise comparisons). Although HDL cholesterol levels rose during follow-up in all groups, they were at a low and borderline low level. We found no statistical difference between the groups (Fig. 3C, p > 0.05 for all pairwise comparisons). The trajectory of plasma triglycerides was only significantly different between the progressing and the J-shape groups (Fig. 2D, P = 0.004 for all pairwise comparisons).

Trajectories of plasma glucose, blood pressure, and lipids in women are shown in Additional file 4: Fig. S2 and Additional file 5: Fig. S3.

## Discussion

In this population-based cohort study of adults aged 20 to 65, we investigated heterogeneity in obesity indices multi-trajectories before the diagnosis of diabetes. We identified three distinct multi-trajectory groups of BMI, WC, and WHR: the progressing group consisted of the majority of patients with a consistent progressive weight gain from overweight to obesity range over 15 years before diabetes incidence; the inverse J-shape with a rapid rise over 10 years before diabetes and rapid decline 3 years before diabetes; the J-shape with a rising trend in obesity indices from 6 years before diabetes diagnosis.

We found that BMI was in the overweight range in all three groups from 15 years before diabetes occurrence and progressed to the obesity range at the time of diabetes onset. Still, the trend of BMI varies between these three groups. Different patterns and inconsistent trends of obesity indices before diabetes development suggest that the cumulative effect of obesity is essential regardless of its pattern. Our finding is consistent with the evidence from Luo et al. study [19], showing that younger age at obesity onset and duration of obesity exposure from young adulthood are associated with diabetes incidents [19]. Zameni et al. [20] found a strong association between cumulative excess weight and cumulative excess waist circumference with incident diabetes among overweight and centrally obese individuals. In another study higher risk of diabetes due to the cumulative effect of obesity was also confirmed [21].

Nano et al. [7] revealed that most diabetic patients got in a progressive overweight group with a progressive weight gain in the overweight range and did not progress to the obesity range. The result of the Whitehall II study also indicated the same pattern for most diabetic patients [8]; however, in our research, the dominant diabetes population progressed to obesity ranging from 10 years before diabetes incidence. It appears that obesity is more common in diabetic patients in our people. In contrast to mentioned studies, in that most of the diabetic population was in the low-risk obesity group, our diabetic patient had a higher BMI and worse trend.

Nano et al. [7] showed two other trajectory groups as progressive weight loss that reaches the BMI in the normal range in diabetes incidence time and persistently high BMI with BMI > 35 from 20 years before diabetes. However, the trajectory of BMI did not show such a contradictory pattern in our study, and all groups had a BMI in the obesity range before diabetes. On the other hand, in the Nano study, the diabetes-free group have a stable BMI of 25 from 20 years ago [7]. Still, in our research, non-diabetic people had a rising trend in BMI from the normal to obesity range over 4 years before the last examination. Although the non-diabetic group also had an increasing trend in obesity indices, the duration of being overweight/obese is shorter than the diabetic occurrence group. So, these findings indicate that, generally, obesity is more common in our population, even in diabetic-free participants. Our study and others revealed that long-term exposure to overweight and obesity is a more critical factor related to diabetes incidence than the trend of obesity. Another study showed that being overweight from childhood has the most association with diabetes incidence among five weight trajectories [22].

We revealed that most diabetes incidence cases were in the group that seems not to be high risk because of not being in the very obese range or rapid weight gain, which can deceive the physician in a preventive training program. In our study, those in the very obese range or rapid weight gain consisted of a small portion of the population. The worse body composite trend in diabetic patients might result from an improper lifestyle and nutritional status. So, the importance of a training program for lifestyle change according to weight status should be emphasized.

We also found that WC and WHR had the same pattern as BMI trajectory; however, in the J-shape group, the rising trend of the WC and WHR index was steeper than BMI. WC and WHR have a better association with diabetes incidence in this group. Although it was established that both BMI and WC were associated with diabetes [23], the evidence suggested that WHR could predict diabetes incidence better than BMI [24]. CHEN et al.’s study showed that as long as WC did not reduce, the risk of diabetes incidents would be high regardless of BMI change [25]. Generally, central obesity indices (WC and WHR ) can predict diabetes incidence better than BMI and weight [26].

We noticed that the trajectories of FBS and 2HPG were the same in these three obesity trajectories, with a steep rise 6 years before diabetes diagnosis. Consistent with our study, other studies showed a rising trend in FBS during time with a sleeper rise from 5 years before diabetes incidence. However, they showed a more variable trend in FBS levels in the weight loss group and a more rapid rise in progressive weight gainers [7, 8].

We also observed that LDL and total cholesterol had a similar rising trend in all three obesity groups and the normal population. Still, the diabetes-free population had a more rapid trend that may result from training individuals at high risk for diabetes and insufficient training in the normal population. Nano et al. [7] also showed no difference in lipid profile between obesity groups. Still, the LDL and total cholesterol trends were higher in the progressive group than in other groups. Besides, in Vistisen’s study, the persistent obese group had higher TG and lower HDL [8].

Interestingly, in our study, the trend of TG followed the same pattern of obesity trajectory of each group, and HDL trajectory has the inverse trajectory pattern of obesity groups. TG and HDL seem to provide the best feature for obesity patterns. Our finding is consistent with some studies showing that BMI positively and negatively relates to TG and HDL, respectively [27–29]. In contrast, patterns of TG and HDL did not follow obesity indices in other studies [7, 8]. Notably, we used a person-centered, multi-trajectory approach that modeled the common progressions of three obesity indices, whereas BMI and WC were evaluated separately in other studies.

Another contribution of this study is that men had more WC, and women were more generally obese. Women’s BMI was in the obesity range from 10 years before diabetes. Still, in men, diabetes occurred 6 years after BMI reached to obesity range. Men and women had WC > 90 cm from 13 to 10 years before diabetes diagnosis. Evidence proposed that body composition and fat distribution are different in men and women, showing that women had more body fat and men had more central fat distribution [30]. Although numerous studies have been suggested a positive association between obesity-related anthropometric indices and diabetes in different sex and age groups, the optimal anthropometric index most closely related to incident diabetes remains controversial. Ge et al. [31] reported that the optimal obesity indices for predicting diabetes are WC in men and WHR and BMI for women aged 18–59 and ≥ 60 years, respectively. In contrast, in Tian et al. [32], WC appeared to be independently associated with an increased risk of diabetes in women. Both BMI and WC showed a positive association with diabetes risk in men.

Finally, it seems that many nondiabetic subjects were having overweight and other components of metabolic syndrome. However, these subjects did not develop diabetes by the time they were in our study, and they might develop it in the future. Besides, a study based on 11 European cohorts showed that 15.7% of nondiabetic men and 14.2% of nondiabetic women had the metabolic syndrome [33].

The main strength of our study is the use of an innovative multi-trajectory model to identify subgroups of participants in a large prospective cohort study with repeated measurements over a substantial follow-up period based on trajectories of multiple obesity indices before diabetes diagnosis. It could help us consider general and central obesity in one model and compare the trajectories of obesity indices in men and women separately. Besides, assessing the obesity indices trajectories over 15 years before the diagnosis of diabetes may provide insights into the association between long-term obesity indices and subsequent diabetes events. Despite these strengths, this study has limitations. Foremost is that the TLGS only contains urban adults in Tehran, which might reduce the generalizability of the results to the mainly rural population. Furthermore, it should be noted that GBTM attempts to classify individuals based on the available data, and they should not be interpreted as intrinsic properties.

## Conclusion

Three patterns of the joint progression of obesity indices before diabetes diagnosis were accompanied by similar patterns of blood glucose and other cardiometabolic risk factors. Most diabetic patients get into the progressive group. All the groups have BMI in the overweight range and progressed to obesity from 6 to 10 years before diabetes. These findings suggest the impact of the increasing trend of obesity indices and other metabolic factors on the incidence of diabetes and emphasize the importance of assessing the metabolic risk factors at each visit.

## Supplementary Information

**Additional file 1: FigureS1.** Flowchart of the participants included in the current study.

**Additional file 2: TableS1.** Latent Class Growth Mixture Models (LCGMM) results of the model fitting process.

**Additional file 3: TableS2.** Parameter estimates for the best fitting 3-class cubic latent class growth mixture model fitted to the lipid profile data.

**Additional file 4: FigureS2.** Trajectories of fasting plasma glucose (A), 2-h plasma glucose (B), systolic blood pressure (C), and diastolic blood pressure (D)for women 53 years of age at time 0 from 15 years before the diagnosis of type2 diabetes or last examination. Trajectories for blood pressure represent men,not on antihypertensive treatment. Lines are the estimated trajectories, andshadows are 95% CIs.

**Additional file 5: FigureS3.** Trajectories of totalcholesterol (A), low-density lipoprotein cholesterol (B), high-densitylipoprotein cholesterol (C), and the logarithm of triglycerides (D)concentrations for women of 53 years of age at time 0 and not on lipid-lowering treatment from 15 years before the diagnosis of type 2 diabetes or last examination. Lines are the estimated trajectories, and shadows are 95% CIs.

## Data Availability

The datasets used during the current study are available from the corresponding author upon reasonable request.

## Abbreviations

BMI: Body mass index
WC: Waist circumference
WHR: Waist-height ratio
FPG: Fasting plasma glucose
2-hPG: 2-Hour plasma glucose
SBP: Systolic blood pressure
DBP: Diastolic blood pressure
TC: Total cholesterol
HDL-C: High-density lipoprotein cholesterol
LDL-C: Low-density lipoprotein cholesterol
TG: Triglycerides
CVD: Cardiovascular disease
ADA: American Diabetes Association
IDF: International Diabetes Federation
TLGS: Tehran Lipid and Glucose Study
BIC: Bayesian information criterion
