# Supplementary figures and images for: Patterns of change in obesity indices and other cardiometabolic risk factors before the diagnosis of type 2 diabetes: two decades follow-up of the Tehran lipid and glucose study
Source: J Transl Med. 2022 Nov 8;20:518. doi: 10.1186/s12967-022-03718-8 (PMC9644604; doi:10.1186/s12967-022-03718-8)

**
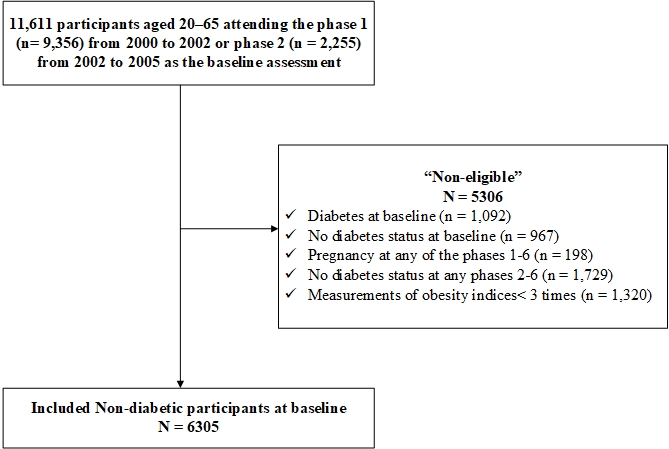
**

**Figure S1.** Flowchart of the participants included in the current study

Supplement: Supplementary file 1 — Additional file 1: FigureS1. Flowchart of the participants included in the current study. [file 12967_2022_3718_MOESM1_ESM.docx]
