# Supplementary material for: Patterns of change in obesity indices and other cardiometabolic risk factors before the diagnosis of type 2 diabetes: two decades follow-up of the Tehran lipid and glucose study
Source: J Transl Med. 2022 Nov 8;20:518. doi: 10.1186/s12967-022-03718-8 (PMC9644604; doi:10.1186/s12967-022-03718-8)
Supplement: Supplementary file 2 — Additional file 2: TableS1. Latent Class Growth Mixture Models (LCGMM) results of the model fitting process. [file 12967_2022_3718_MOESM2_ESM.docx]

**Table S1.** Latent Class Growth Mixture Models (LCGMM) results of the model fitting process.

| No. of latent  classes | Polynomial  degree | Log-Lik | BIC | % Participants per group | Average posterior probabilities |
| --- | --- | --- | --- | --- | --- |
| 1 | Linear | -15330.66 | 30885.16 | - | - |
|  | Quadratic | -15264.1 | 30765.61 | - | - |
|  | Cubic | -15260.3 | 30771.58 | - | - |
| 2 | Linear | -15325.36 | 30894.92 | 13.5**/**86.5 | 0.74**/**0.88 |
|  | Quadratic | -15138.44 | 30541.43 | 16.9**/**81.1 | 0.79**/**0.89 |
|  | Cubic | -15108.51 | 30501.93 | 13.1**/**86.9 | 0.82**/**0.94 |
| 3 | Linear | -15291.65 | 30847.85 | 13.6**/**1.0**/**85.4 | 0.74**/**0.93**/**0.87 |
|  | Quadratic | -15072.99 | 30437.67 | 85.4**/**4.3**/**10.3 | 0.89**/**0.84**/**0.77 |
|  | **Cubic** | **-15019.86** | **30358.54** | **83.2/5.9/10.9** | **0.91/0.85/0.80** |
| 4 | Linear | -15315.01 | 30915.26 | 1.1**/**13.8**/**59.5**/**25.5 | 0.89**/**0.81**/**0.78**/**0.77 |
|  | Quadratic | -15063.88 | 30446.94 | 15.8**/**4.0**/**79.1**/**1.0 | 0.73**/**0.83**/**0.83**/**0.85 |
|  | Cubic | -14991.73 | 30336.61 | 73.1**/**6.2**/**2.5**/**18.2 | 0.78**/**0.84**/**0.86**/**0.71 |
| Reported are: the number of trajectory groups considered, the polynomial form of the model, the maximum Log-Likelihood (Log-Lik), the Bayesian Information Criterion (BIC), the posterior classification of subjects in each class (%), the average of posterior probabilities (AvePP) in each group. The best-fitting model is highlighted in bold characters. | | | | | |
