# Supplementary material for: Patterns of change in obesity indices and other cardiometabolic risk factors before the diagnosis of type 2 diabetes: two decades follow-up of the Tehran lipid and glucose study
Source: J Transl Med. 2022 Nov 8;20:518. doi: 10.1186/s12967-022-03718-8 (PMC9644604; doi:10.1186/s12967-022-03718-8)
Supplement: Supplementary file 3 — Additional file 3: TableS2. Parameter estimates for the best fitting 3-class cubic latent class growth mixture model fitted to the lipid profile data. [file 12967_2022_3718_MOESM3_ESM.docx]

**Table S2.** Parameter estimates for the best fitting 3-class cubic latent class growth mixture model fitted to the lipid profile data.

| Polynomial term | Class | Coefficient | SE | Wald | p-value |
| --- | --- | --- | --- | --- | --- |
| Intercept | Progressing | 0† |  |  |  |
|  | Inverse J-shape | -0.227 | 0.173 | -1.313 | 0.189 |
|  | J-shape | 0.135 | 0.144 | 0.934 | 0.350 |
| Time | Progressing | 0.063 | 0.017 | 3.644 | <0.001 |
|  | Inverse J-shape | -0.480 | 0.042 | -11.423 | <0.001 |
|  | J-shape | 0.355 | 0.051 | 6.899 | <0.001 |
| Time^2/10 | Progressing | 0.053 | 0.023 | 2.289 | <0.001 |
|  | Inverse J-shape | -0.906 | 0.069 | -13.143 | 0.022 |
|  | J-shape | 0.392 | 0.080 | 4.886 | <0.001 |
| Time^3 /20 | Progressing | 0.007 | 0.002 | 3.456 | <0.001 |
|  | Inverse J-shape | -0.072 | 0.006 | -11.349 | <0.001 |
|  | J-shape | 0.026 | 0.007 | 3.869 | <0.001 |
| Age53 |  | 0.118 | 0.030 | 3.869 | <0.001 |
| Phase |  | 0.082 | 0.033 | 2.474 | 0.013 |
| Sex |  | -0.121 | 0.076 | -1.592 | 0.111 |
| Sex* time |  | -0.077 | 0.017 | -4.579 | <0.001 |
| Sex*time^2 |  | -0.077 | 0.028 | -2.734 | 0.006 |
| Sex*time^3 |  | -0.005 | 0.003 | -1.746 | 0.081 |
| Contrasts on sex (p<0.00001) | |  |  |  |  |
| BMI |  | -0.992 | 0.047 | -21.059 | <0.001 |
| WC |  | -0.028 | 0.028 | -1.022 | 0.307 |
| WHR |  | 1.020 | 0.051 | 19.998 | <0.001 |

SE, standard error; BMI, body mass index; WC, waist circumference; WHR, waist to hip ratio.

†Not estimated, the mean intercept in the first class is constrained to 0.
