# Supplementary material for: Patterns of change in obesity indices and other cardiometabolic risk factors before the diagnosis of type 2 diabetes: two decades follow-up of the Tehran lipid and glucose study
Source: J Transl Med. 2022 Nov 8;20:518. doi: 10.1186/s12967-022-03718-8 (PMC9644604; doi:10.1186/s12967-022-03718-8)
Supplement: Supplementary file 4 — Additional file 4: FigureS2. Trajectories of fasting plasma glucose (A), 2-h plasma glucose (B), systolic blood pressure (C), and diastolic blood pressure (D)for women 53 years of age at time 0 from 15 years before the diagnosis of type2 diabetes or last examination. Trajectories for blood pressure represent men,not on antihypertensive treatment. Lines are the estimated trajectories, andshadows are 95% CIs. [file 12967_2022_3718_MOESM4_ESM.docx]

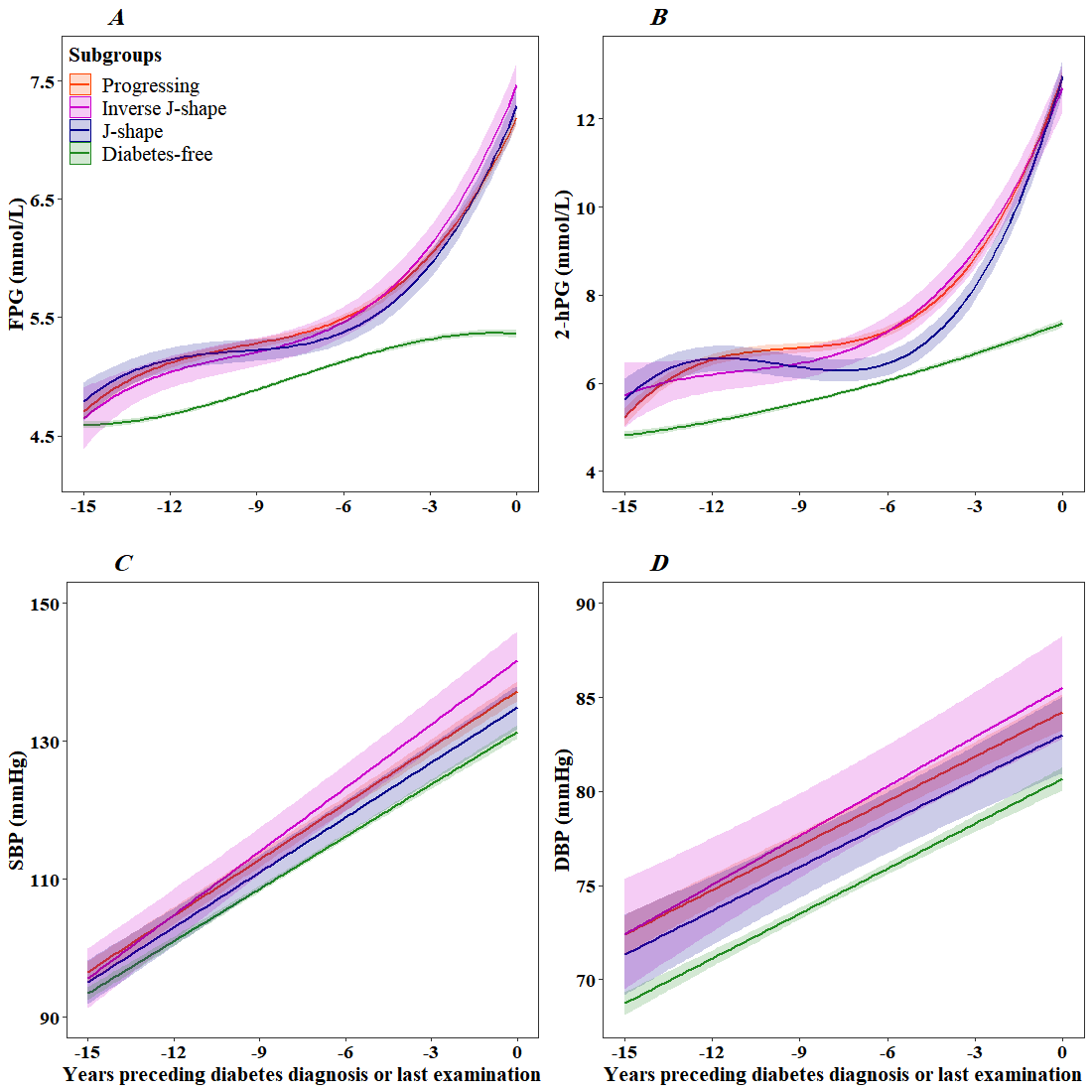


**Figure S2.** Trajectories of fasting plasma glucose (A), 2-hour plasma glucose (B), systolic blood pressure (C), and diastolic blood pressure (D) for women 53 years of age at time 0 from 15 years before the diagnosis of type 2 diabetes or last examination. Trajectories for blood pressure represent men, not on antihypertensive treatment. Lines are the estimated trajectories, and shadows are 95% CIs.
