# Supplementary material for: Patterns of change in obesity indices and other cardiometabolic risk factors before the diagnosis of type 2 diabetes: two decades follow-up of the Tehran lipid and glucose study
Source: J Transl Med. 2022 Nov 8;20:518. doi: 10.1186/s12967-022-03718-8 (PMC9644604; doi:10.1186/s12967-022-03718-8)
Supplement: Supplementary file 5 — Additional file 5: FigureS3. Trajectories of totalcholesterol (A), low-density lipoprotein cholesterol (B), high-densitylipoprotein cholesterol (C), and the logarithm of triglycerides (D)concentrations for women of 53 years of age at time 0 and not on lipid-lowering treatment from 15 years before the diagnosis of type 2 diabetes or last examination. Lines are the estimated trajectories, and shadows are 95% CIs. [file 12967_2022_3718_MOESM5_ESM.docx]

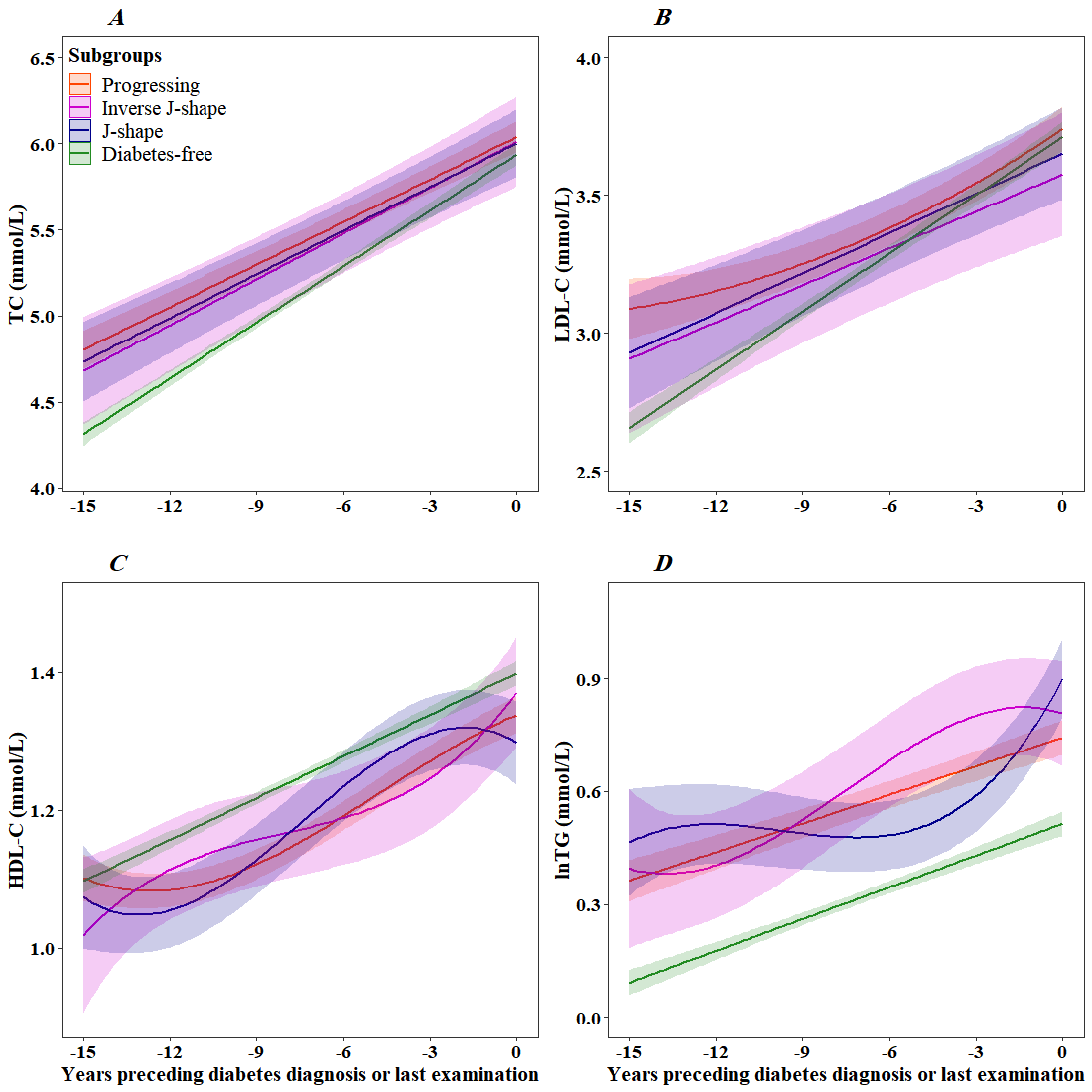


**Figure S3.** Trajectories of total cholesterol (A), low-density lipoprotein cholesterol (B), high-density lipoprotein cholesterol (C), and the logarithm of triglycerides (D) concentrations for women of 53 years of age at time 0 and not on lipid-lowering treatment from 15 years before the diagnosis of type 2 diabetes or last examination. Lines are the estimated trajectories, and shadows are 95% CIs.
